# 

**Published:** 1865-01

**Authors:** 


					﻿THE
Dental Register
OF THE west’
Edited and Published by J. Taft.
JANUARY, 1865.
MONTHLY:
Three lyollars per An nit m, in Adennee.
CINCINNATI:
WRIGHTSON CO., Printers, 167 WALNUT STREET.
.r. TAFT, Jienfisf, 172 Jtftce Sfi-eef.
				

